# Balance of Population

**Published:** 1860-05

**Authors:** 


### BALANCE OF POPULATION.

In Newtown, Mass., one person in every seven, of eight thousand souls is Irish; at the same time, half of all the births were of Irish children; that is, one Irishman is equal to six Americans in populating power. It is stated that in Boston, the births from Irish parents are more numerous than American. At this rate, we will soon be extinguished, like the Sandwich Islander and the Indian. But there is an antagonizing influence always at work. The Irish are day-laborers; the Americans are well to do, hence, live ten or fifteen years longer; in addition, the inherent love of liquor, an appetite which seems to be born with them, works an early death; another still greater cause of mortality, is their domestic habits; as a class, they live in hovels, unfit even for the beasts of the field; this, with their notorious improvidence, aids in bringing about the general result of a greater proportional mortality.

The same general rule holds good as to rich and poor; the laboring poor have the most children, as in the days before Moses and Aaron; at the same time, their greater exposures, hardships, privations, excesses, and sorrows equalize populatins.

